# The Role of OCTA and Microperimetry in Revealing Retinal and Choroidal Perfusion and Functional Changes Following Silicone Oil Tamponade in Rhegmatogenous Retinal Detachment: A Narrative Review

**DOI:** 10.3390/diagnostics15192422

**Published:** 2025-09-23

**Authors:** Dan-Grigore Dunca, Simona-Delia Nicoară

**Affiliations:** 1 Department of Ophthalmology, “Iuliu Hațieganu’’ University of Medicine and Pharmacy, 8 Victor Babeș Street, 400012 Cluj-Napoca, Romania; simonanicoara1@gmail.com; 2 Clinic of Ophthalmology, Emergency County Hospital, 3–5 Clinicilor Street, 400006 Cluj-Napoca, Romania

**Keywords:** rhegmatogenous retinal detachment, silicone oil tamponade, retinal sensitivity, retinal perfusion

## Abstract

**Background**: Rhegmatogenous retinal detachment (RRD), the most common type of retinal detachment, requires prompt surgery to reattach the retina and avoid permanent vision loss. While surgical treatment is adapted to each individual case, one frequent option is pars plana vitrectomy (PPV) with silicone oil (SO) tamponade. Despite achieving anatomical success (complete retinal attachment), concerns persist regarding potential microvascular alterations in the retina and choroid, with a negative impact on visual function. Optical coherence tomography angiography (OCTA) allows detailed, in-depth imaging of retinal and choroidal circulation, whereas microperimetry makes it possible to accurately assess macular function. This review aims to strengthen the existing evidence on vascular and functional alterations at the macular level after SO tamponade in cases of RRD. **Methods**: A narrative review was conducted using a structured approach, utilizing a PubMed search from January 2000 up to April 2025. Twenty-three studies on OCTA and microperimetry after SO tamponade for RRD were included. Data on vessel densities, choroidal vascular index (CVI), foveal avascular zone (FAZ) size, and retinal sensitivity were extracted and qualitatively analyzed. **Results**: Studies consistently reported a reduction in the vessel density within the superficial capillary plexus (SCP) under SO tamponade, with partial but incomplete reperfusion post-removal. Choroidal perfusion and CVI were also decreased, exhibiting a negative correlation with the duration of SO tamponade. Microperimetry demonstrated significant reductions in retinal sensitivity (~5–10 dB) during SO tamponade, which modestly improved (~1–2 dB) following removal but generally remaining below normal levels. **Conclusions**: SO tamponade causes substantial retinal and choroidal vascular impairment and measurable macular dysfunction, even after anatomical reattachment of the retina. It is recommended to perform early SO removal (~3–4 months) and implement routine monitoring by OCTA and microperimetry with the aim of optimizing patient outcomes. Future research should focus on investigating protective strategies and enhancing visual rehabilitation following RRD repair.

## 1. Introduction

Rhegmatogenous retinal detachment (RRD) is the most common type of retinal detachment, defined by the separation of the neurosensory retina from the underlying retinal pigment epithelium, due to one or more retinal holes or tears that permit vitreous fluid to enter the subretinal space [1,2]. The primordial event is represented by vitreous liquefaction, followed by either chronic, or acute posterior vitreous detachment during which one or more breaks can occur. If not operated promptly, it causes permanent visual loss [3]. Epidemiologically, the annual incidence of RRD has been reported to vary between 10 and 18 cases per 100,000 individuals, highlighting its considerable impact on the visual function and quality of life, particularly when the macula becomes detached [4,5]. Established risk factors are advanced age, myopia, ocular trauma, prior cataract surgery and hereditary predisposition, underscoring the critical importance of prompt diagnosis and therapeutic intervention [5,6].

Pars plana vitrectomy (PPV) has become the primary surgical procedure for treating RRD. This technique involves removing the vitreous body to relieve any vitreoretinal traction and enable secure closure of retinal breaks. Intraocular tamponades are used at the end of the procedure, silicone oil (SO) being particularly preferred in complex RRD, proliferative vitreoretinopathy (PVR) and giant retinal tears, due to its long-lasting tamponading effect and dependable stabilization qualities [7,8]. SO tamponade greatly improves anatomical success by sustaining stable retinal reattachment over a long period of time [9,10]. Its high viscosity and inert nature allow it to remain safely inside the eye for extended periods, enhancing long-term retinal adhesion and lowering the risk of re-detachment. Therefore, SO has become an essential part of managing complex RRD cases [11].

Although effective in complex RRDs, SO is associated with several potential complications that can impact retinal and choroidal health, as well as visual outcomes. Vascular alterations are frequently reported, with studies demonstrating a reduction in vessel density (VD) within retinal capillary plexi and the choriocapillary, possibly resulting from prolonged mechanical compression or biochemical effects of SO on microvascular structures [10,11]. Structural modifications include the thinning of retinal layers, notably the retinal nerve fiber layer and inner retinal structures, which may indicate long-term ischemic or mechanical damage. Clinically significant visual deficits can persist even after successful anatomical repair, appearing as reduced visual acuity, impaired contrast sensitivity and metamorphopsia, which are often due to subtle microstructural and microvascular damage [12,13].

Advanced diagnostic tools, notably Optical Coherence Tomography Angiography (OCTA) and microperimetry, have significantly improved the detection and characterization of subtle anatomical and functional retinal changes following SO tamponade in cases of RRD. OCTA is a non-invasive imaging technique that provides in-depth visualization of retinal and choroidal microvascular structures [14,15]. Additionally, microperimetry complements OCTA by objectively assessing retinal sensitivity and fixation, offering a comprehensive functional evaluation of the retina [16]. This approach is especially useful for assessing potential visual rehabilitation, as it can detect subtle functional deficits that are not evident through standard visual acuity tests [17]. Collectively, these diagnostic techniques give clinicians important insights into postoperative microvascular and functional changes, improving prognostic accuracy and guiding targeted postoperative treatment strategies.

SO tamponade can have a negative impact on retinal microvasculature and function following RRD repair. OCTA investigations have documented a decline in macular VD during SO tamponade. In cases of macula-off RRD, both retinal thickness and perfusion remain markedly diminished even after the removal of the oil. Although some perfusion recovery is noted post-removal, particularly in macula-on cases, peripapillary perfusion deficits may endure [18]. From a functional perspective, SO is associated with diminished retinal sensitivity; microperimetry reveals a reduction in foveal sensitivity under SO compared to gas tamponade [19]. Encouragingly, retinal sensitivity and visual acuity frequently show improvement following SO removal, implying a degree of reversibility in the toxicity [20,21]. However, current research is subject to limitations. Most studies involve small cohorts with brief follow-up durations, thereby limiting statistical robustness [21]. Additionally, heterogeneity in detachment status (macula-on vs. macula-off) and variations in imaging protocols further complicate interpretation, highlighting the necessity for cautious conclusions.

This review aims to synthesize evidence from OCTA and microperimetry studies on eyes post-SO tamponade for RRD, to clarify microvascular and functional outcomes. We collated findings on macular microcirculation changes (e.g., capillary density loss, alterations in the foveal avascular zone–FAZ) and visual function deficits (e.g., reduced retinal sensitivity or central scotomas) following the use of SO. We outlined the scope of current research and emphasized existing gaps, noting that the literature remains relatively scarce and sometimes contradictory regarding SO-related retinal changes [18]. Our goal was to highlight the importance of standardized research protocols, including uniform OCTA metrics, microperimetry methods, and timing, to better understand the effects of SO on the retina and to inform future management.

## 2. Materials and Methods

### 2.1. Data Sources and Search Strategy

This narrative review was conducted with a structured search and selection process. Although not designed as a systematic review, several PRISMA-inspired elements—such as an explicit search strategy, predefined eligibility criteria, and transparent reporting of study selection—were incorporated to enhance transparency, reproducibility, and methodological rigor. A comprehensive PubMed search was performed to identify studies evaluating retinal and choroidal perfusion, as well as functional outcomes, following SO tamponade in eyes operated for RRD. The search strategy, detailed in Supplementary Material S1, included relevant keywords and Medical Subject Headings (MeSH), such as “rhegmatogenous retinal detachment,” “silicone oil tamponade,” “retinal perfusion,” “choroidal perfusion,” “optical coherence tomography angiography,” and “microperimetry.” The search covered all available PubMed records up to April 2025, without restrictions on the lower date limit, thereby capturing both recent and older studies. Only studies published in English were considered eligible.

Initially, titles and abstracts of retrieved articles were screened to exclude unrelated publications. After removing duplicates, 156 potentially relevant records were selected. Additionally, the reference lists of identified studies were manually examined to ensure comprehensive coverage and inclusion of pertinent studies that might have been missed by the electronic search.

### 2.2. Eligibility Criteria

Studies were selected based on clearly defined inclusion and exclusion criteria.

Eligible studies had to be original research articles employing observational or longitudinal designs (either prospective or retrospective), specifically involving patients with RRD who underwent PPV with SO tamponade. Included studies reported ocular perfusion or functional outcomes, emphasizing alterations in retinal microvascular parameters (e.g., capillary VD, FAZ), choroidal perfusion or thickness, peripapillary perfusion, or retinal functional performance measured by microperimetry, following SO tamponade and subsequent to SO removal, where applicable. All studies were required to involve human subjects and be published in peer-reviewed English-language journals.

Exclusion criteria encompassed articles that did not present original research, such as narrative reviews, systematic reviews, meta-analyses, case reports, conference abstracts, letters, or editorials. Moreover, studies not published in English or those that did not specifically investigate retinal or choroidal perfusion or functional visual outcomes associated with SO tamponade in the context of RRD were excluded. To prevent data duplication, in the situation of multiple publications reported on the same patient cohort, only the most recent or comprehensive study was included.

These explicitly delineated criteria were consistently applied throughout the screening and selection process to guarantee the inclusion of only the most pertinent and high-quality evidence.

### 2.3. Quality Assessment and Data Extraction

**Figure 1:**
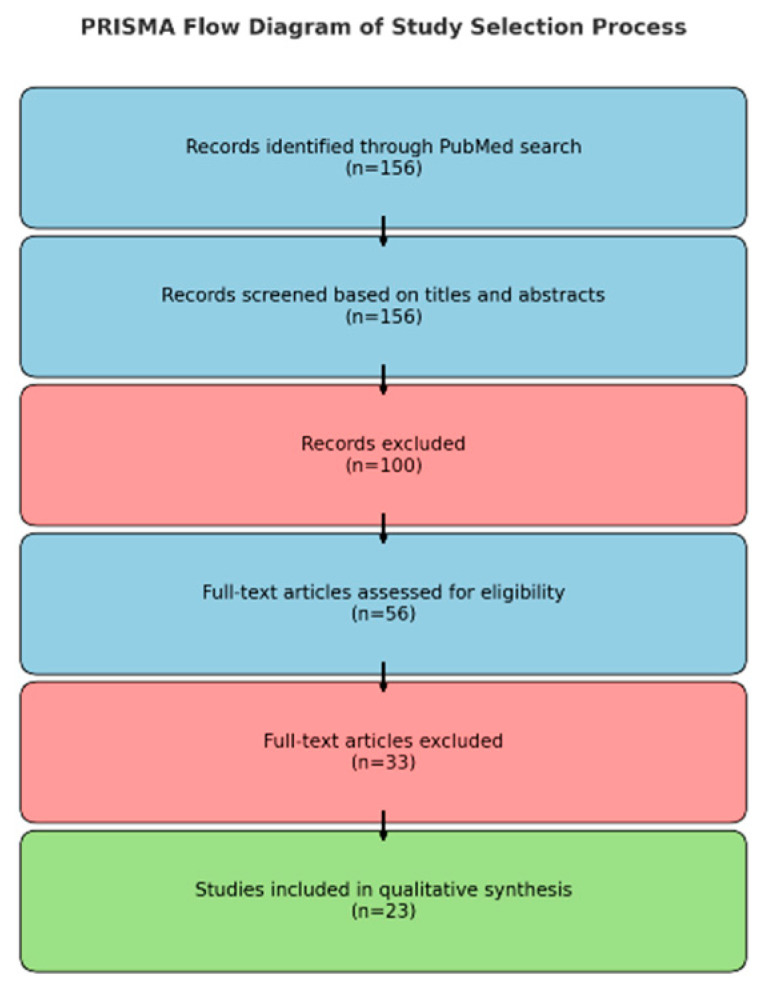
Selection process of studies included in the qualitative review.

All records identified through the database search were screened in accordance with predefined eligibility criteria. Although this review was conducted as a narrative synthesis, we incorporated several elements of the PRISMA framework—including dual independent screening, inclusion/exclusion criteria, and study selection flow—to enhance methodological transparency. Of the 156 records initially retrieved, 53 articles were assessed in full text, and 23 studies met the inclusion criteria and were incorporated into the qualitative synthesis (Figure 1, Selection process of studies included in the qualitative review).

Of these 23 studies, 11 primarily examined retinal vascular changes, 5 focused on choroidal vascular changes, and 2 evaluated peripapillary vascular changes, with 1 of these also providing data relevant to retinal vascular changes. Additionally, 7 studies assessed retinal sensitivity and functional outcomes using microperimetry, among which 1 study also reported retinal vascular data.

Data from each study were extracted by two independent reviewers working in parallel to minimize errors. Extracted data included study design details (e.g., prospective vs. retrospective), sample characteristics (number of eyes/patients, demographic and clinical features), specifics of the intervention (duration of SO tamponade, timing of SO removal, if reported), imaging modalities, key outcome measures (e.g., OCTA parameters, such as VD in superficial and deep capillary plexuses, choriocapillaris perfusion indexes, peripapillary capillary density, or retinal sensitivity assessed via microperimetry), and primary findings relevant to retinal or choroidal perfusion and visual function. The two reviewers compared the extracted data for consistency, resolving minor discrepancies by jointly reviewing the original articles.

Although this review was conducted as a narrative synthesis, we performed a structured evaluation of the methodological quality of the included studies to improve transparency. All 23 studies focused on retinal and/or choroidal perfusion and functional changes after SO tamponade for RRD and were observational in design (retrospective cohorts, prospective cohorts, or cross-sectional/before–after series). We assessed the risk of bias using the Newcastle–Ottawa Scale (NOS), applying the cohort version and adapting it for cross-sectional or before–after designs. Two reviewers conducted the assessment independently and resolved disagreements by consensus. NOS scores (maximum 9 stars) were categorized as follows: low risk (7–9), moderate risk (5–6), and high risk (≤4). A detailed study-level assessment is provided in Supplementary Table S2.

By implementing a standardized screening and data extraction process with independent reviewers and by incorporating selected PRISMA-inspired elements, we aimed to enhance the reliability, reproducibility, and clarity of this narrative review, ensuring a comprehensive and transparent synthesis of the current evidence on retinal and choroidal perfusion and functional changes after SO tamponade in RRD.

## 3. Results

### 3.1. Macular Retinal Vascular Changes

Multiple studies consistently report a reduction in VD within the superficial capillary plexus (SCP) and inner retinal thickness (IRT) following SO tamponade, with limited recovery often observed after the tamponade was removed. An overview of the studies assessing FAZ, VD, and retinal thickness after RRD surgery is provided in Table 1.

#### 3.1.1. Superficial Capillary Plexus Vessel Density Changes

The reviewed studies reported varied outcomes regarding superficial SCP-VD following SO tamponade. Several investigations identified significant reductions in SCP-VD postoperatively.

In Jiang et al., SCP-VD was lower at 2 weeks vs. fellow eyes (38.5% vs. 48.9%, *p* < 0.001), the between-eye difference was no longer significant at 12 weeks (48.5% vs. 49.3%, *p* = 0.334), then decreased again by 16 weeks (45.9% vs. 48.9%, *p* = 0.004) during ongoing SO tamponade [22].

Gironi et al. reported a persistent reduction in SCP-VD even at 12 months after SO removal, negatively correlated with tamponade duration (r = −0.52) [23]. Liu et al. also described significantly lower parafoveal SCP-VD in SO-treated eyes compared to fellow eyes at the final follow-up at least 30 months after PPV and following SO removal (39.3% vs. 45.5%, *p* < 0.001) [24]. Fang et al. observed that parafoveal SCP-VD was lower in eyes treated with SO compared to air tamponade controls at 1 month (43.8% vs. 47.7%; *p* = 0.0403), and this difference persisted at three months (43.9% vs. 50.4%; *p* = 0.001). No intra-SO improvement was reported [25]. Salehi et al. confirmed a significant reduction in SCP-VD before and after SO removal, maintaining a lower density compared to fellow eyes at 3 months (37.4% vs. 43.4%, *p* = 0.02) [26].

Conversely, several studies reported minimal or no SCP changes. Lee et al. found no significant difference in SCP-VD (34.1% vs. 34.2%, *p* = 0.873) between SO-treated and fellow eyes at three months following SO removal [27]. Lee and Park noted overall stable SCP-VD following SO removal, except for a localized deficit in the nasal parafoveal area (*p* = 0.028) [28]. Nassar et al. observed no statistically significant differences in SCP-VD before and after SO removal across all macular regions (all *p* > 0.3) [20]. Likewise, Ya et al. reported comparable SCP densities between formerly SO-treated eyes and healthy fellow eyes at 3 months post-removal (*p* = 0.873) [29].

#### 3.1.2. Deep Capillary Plexus Vessel Density Changes

Studies evaluating VD within the deep capillary plexus (DCP) following SO tamponade have yielded diverse results. Jiang et al. observed that parafoveal DCP-VD was significantly reduced at 2–8 weeks after PPV with SO (all *p* < 0.001 vs. fellow eyes), became statistically indistinguishable at 12 weeks (53.54% vs. 54.33%; *p* = 0.247), and decreased again by 16 weeks (51.49% vs. 54.28%; *p* = 0.007) during SO tamponade [22]. Likewise, Lee and Park reported a significant decrease in average parafoveal DCP-VD at 3 months after SO removal, particularly in the nasal and temporal quadrants (*p* = 0.026–0.033), noting that the reduction was more pronounced in macula-off cases [28]. Furthermore, Lee et al. documented persistent significant deficits in DCP at 3 months following SO removal, with greater vessel loss associated with longer retention times of SO [27].

Conversely, several studies identified preserved DCP perfusion. Liu et al. reported no significant differences in parafoveal DCP-VD between the SO-treated eyes and their fellow eyes (51.16% vs. 51.57%, *p* = 0.807), evaluated at least 30 months after PPV and following SO removal [24]. Nassar et al. similarly documented stable DCP-VD pre- and post-SO removal, noting minor, statistically insignificant increases [20]. Roohipoor et al. reported slight improvements in foveal DCP-VD at one month (*p* = 0.022), although parafoveal DCP-VD remained unchanged before SO removal [30]. Salehi et al. reported no significant change in DCP vessel density up to 3 months after silicone-oil removal, with persistent deficits versus fellow eyes limited to the temporal parafovea/perifovea [26]. Fang et al. reported stable DCP-VD over three months without substantial changes during the SO tamponade, and Ya et al. confirmed no significant differences in DCP-VD between SO-treated and fellow eyes across multiple macular sectors (*p* = 0.136–0.915), during the SO tamponade, prior to its removal [25,29].

#### 3.1.3. FAZ Metrics

Several studies have assessed the impact of SO tamponade on FAZ metrics, yielding mixed results. Notably, Lee and Park documented significant enlargement of the FAZ following SO tamponade, observing increased FAZ areas in both the SCP (SCP; 0.28 ± 0.14 mm^2^ vs. 0.26 ± 0.13 mm^2^, *p* = 0.043) and the DCP (DCP; 0.56 ± 0.15 mm^2^ vs. 0.27 ± 0.12 mm^2^, *p* = 0.002), particularly in cases with macular detachment [28]. Additionally, Lee et al. reported a significant expansion of the FAZ in the DCP (0.73 ± 0.32 mm^2^ vs. 0.60 ± 0.22 mm^2^, *p* < 0.001), which correlated with the duration of SO tamponade (*p* = 0.034). Conversely, the FAZ in the SCP remained unaffected [27].

Numerous studies have documented minimal or no significant alterations in the FAZ following SO tamponade. Liu et al. reported no noteworthy difference in FAZ area between SO-treated eyes and the contralateral eyes (0.267 ± 0.110 mm^2^ vs. 0.339 ± 0.088 mm^2^, *p* = 0.061) [24]. Nassar et al. observed stable FAZ metrics before and after SO removal (0.31 ± 0.47 mm^2^ vs. 0.28 ± 0.17 mm^2^, *p* = 0.758) [20]. Similarly, Roohipoor et al. identified no significant changes at any postoperative intervals [30]. Salehi et al. affirmed the stability of FAZ area and perimeter throughout their follow-up period (*p* = 0.6), evaluated after SO removal [26]. Fang et al. found no differences between SO and air tamponade groups, and Ya et al. reported comparable FAZ areas in SO-treated eyes and fellow eyes (0.31 ± 0.12 mm^2^ vs. 0.25 ± 0.12 mm^2^, *p* = 0.397), prior to SO removal [25,29].

#### 3.1.4. Retinal Thickness

Studies evaluating alterations in retinal thickness following SO tamponade have yielded varied results. Jiang et al. found that, before SO removal, foveal macular thickness (FMT) in SO-filled eyes did not change significantly over time (ANOVA *p* = 0.303) but remained consistently thinner than the fellow eye from week 4 through week 16 (210.37 ± 43.07 µm at week 16; *p* = 0.001 vs. fellow eye) [22]. Liu et al. reported a significant decrease in IRT in eyes treated with SO compared to the contralateral healthy eyes (107.94 ± 15.21 µm vs. 128.38 ± 11.06 µm, *p* < 0.001), with no substantial changes observed in outer or total retinal thickness [24].

Conversely, Nassar et al. observed a significant increase in central foveal thickness (CFT), from 254.90 ± 29.63 µm preoperatively to 265.50 ± 30.40 µm postoperatively (*p* = 0.002), indicating partial structural recovery following SO removal [20].

Meanwhile, Salehi et al. found that macular thickness remained stable during a 3 month follow-up period after SO removal, with no statistically significant changes in total macular thickness (330.6 ± 154.96 µm preoperatively, measured one week prior to SO removal, vs. 310.8 ± 124.68 µm three months post-removal, *p* = 0.46) [26].

### 3.2. Peripapillary Vascular Changes

Peripapillary vascular alterations subsequent to SO tamponade demonstrate significant variability in VD and retinal nerve fiber layer thickness (RNFLT). In a disk-scan OCTA study, Jiang et al. reported that the global radial peripapillary capillary (RPC) VD was lower than that observed in fellow eyes at two weeks postoperatively following PPV with SO tamponade (t = −4.851, *p* < 0.001). The VD increased at four weeks and subsequently decreased, with no statistically significant change observed within the eye between four and twelve weeks (ANOVA F = 1.046, *p* = 0.377; 4 vs. 12 weeks *p* = 0.082). At twelve weeks, the superior hemifield exhibited lower RPC-VD compared to the inferior hemifield (t = −2.844, *p* = 0.010). RNFLT demonstrated a decrease over time; however, the overall change was not statistically significant (ANOVA F = 1.312, *p* = 0.276). Notably, the superior hemifield was thinner than the inferior at twelve weeks (t = −2.222, *p* = 0.037). Furthermore, RNFLT showed a correlation with RPC-VD at all time points (all *p* < 0.05) [31].

Salehi et al. documented persistently diminished peripapillary VD parameters even three months following SO removal, with no notable vascular recovery. RNFLT remained stable throughout the follow-up period, suggesting that the observed vascular impairment persisted without associated anatomical recovery [26].

### 3.3. Choroidal Vascular Changes

Studies assessing choroidal vascular modifications subsequent to SO tamponade exhibit significant variability, particularly concerning the choroidal vascular index (CVI), subfoveal choroidal thickness (SFCT), and choriocapillaris perfusion. Table 2 summarizes the evaluation of SFCT and choriocapillaris changes after RRD surgery.

Chen et al. documented a substantial decrease in CVI during SO tamponade (57.6% vs. 60%, *p* < 0.0001), with a partial recovery following removal (59.5% vs. 60.3%, *p* = 0.031). Correspondingly, the luminal area experienced a significant reduction during SO tamponade (*p* = 0.007), with subsequent recovery post-removal (*p* = 0.028). The stromal area, total choroidal area, and overall choroidal thickness remained unchanged (all *p* > 0.05). The magnitude of CVI reduction was positively associated with the duration of SO tamponade (*p* = 0.035) [32].

Karasu et al. observed significant progressive reductions in SFCT correlating with the duration of SO tamponade, particularly within the 9–18 months period, which exhibited a decrease of 51.50 μm. Partial recovery of SFCT was observed following the removal of the tamponade, especially after extended durations [33].

Supporting these observations, Karimi et al. identified significant SFCT thinning, particularly pronounced in SO durations exceeding nine months (a reduction of 62.1 µm, *p* < 0.001), with minimal recovery post-removal (~2 µm). A significant negative correlation was observed between SO tamponade duration and SFCT (r = 0.398, *p* = 0.002) [34].

Likewise, Mirza et al. documented a notable decrease in SFCT one month following SO removal (from 294.1 µm to 264.2 µm, *p* = 0.03), which demonstrated a strong correlation with the duration of SO tamponade (ρ = 0.537, *p* = 0.032) [35].

Prasuhn et al. reported significant alterations in choroidal perfusion after the removal of SO. Specifically, they noted a statistically significant increase in choriocapillaris perfusion from 45.09% to 45.8% (*p* = 0.0013), whereas deeper choroidal layers exhibited decreased perfusion, with Sattler’s layer decreasing from 59.19% to 57.72% (*p* = 0.034), and Haller’s layer from 62.37% to 60.53% (*p* = 0.0402). Interestingly, SFCT remained stable despite these perfusion changes. These measurements were conducted one week before and four weeks after SO removal [36].

### 3.4. Functional Retinal Changes Evaluated by Microperimetry

Research evaluating retinal function through microperimetry subsequent to SO tamponade and removal consistently documents notable changes in retinal sensitivity and fixation stability. Functional outcomes, including microperimetry and BCVA after RRD surgery with SO tamponade, are presented in Table 3.

Delolme et al. observed a mean central 12° retinal sensitivity of 14.15 ± 2.15 dB and a central 4° foveal sensitivity of 14.04 ± 3.32 dB following RRD repair during the period of SO tamponade, prior to its removal. Fixation stability was preserved in 80% of patients. A statistically significant correlation was identified between diminished retinal sensitivity and photoreceptor abnormalities (*p* = 0.011) [37].

Dou et al. demonstrated notable enhancements in retinal sensitivity subsequent to the removal of SO. The central 2° sensitivity increased from 19.85 ± 5.41 dB to 23.27 ± 3.64 dB (*p* < 0.05), while fixation stability within the same area improved from 72.23% to 79.25% (*p* < 0.05) [38].

Nagpal et al. documented considerable improvements in retinal sensitivity within the central 6° macular region following SO removal, with values significantly increasing from 766.95 ± 173.29 dB to 863.8 ± 181.08 dB (*p* < 0.0001). Nevertheless, changes in visual acuity were minimal and did not reach statistical significance [39].

Nassar et al. (2024) reported notable enhancements in overall retinal sensitivity following SO removal, with values rising from 5.04 ± 2.06 dB to 6.38 ± 2.34 dB (*p* < 0.001) [20]. Statistically significant improvements were observed across the inner, middle, and outer retinal rings (*p* < 0.001–0.002) [20].

Similarly, Nassar et al. (2019) highlighted notable improvements in retinal sensitivity following SO removal in groups with short (<3 months) and longer (3–6 months) tamponade durations [21]. Overall, retinal sensitivity increased significantly from 5.68 ± 2.00 dB to 8.70 ± 2.56 dB (*p* = 0.008) and from 7.00 ± 2.55 dB to 9.83 ± 3.36 dB (*p* = 0.002), respectively. Visual acuity improvements were also statistically significant in both groups [21].

In contrast, Scheerlinck et al. (2016) characterized SO–related visual loss through the identification of a significant central scotoma, wherein the median sensitivity notably diminished at the central points (0.0 dB at 0° and 1°), with peripheral sensitivity remaining relatively preserved (13.9 dB) [40]. Significantly, visual impairment occurred both during SO tamponade and subsequently to SO removal, with the duration of tamponade recognized as a substantial risk factor (*p* = 0.005) [40].

Furthermore, Scheerlinck et al. (2018) demonstrated a statistically significant reduction in central retinal sensitivity following SO tamponade relatively to gas tamponade, observed in both macula-on (11.8 dB vs. 15.6 dB, *p* = 0.003) and macula-off cases (11.6 dB vs. 15.0 dB, *p* = 0.037) [19].

### 3.5. Potential Recovery After Silicone Oil Removal

Research assessing the restoration of retinal structure and vasculature following the removal of SO has shown variable outcomes, thereby indicating differing capacities for recovery.

Several studies have documented significant structural and vascular enhancements following SO removal. Hou et al. observed a marked recovery in macular SVD and superficial perfusion density (SPD) starting one month after SO removal and persisting at three months (*p* < 0.001), with these improvements positively correlating with enhancements in visual acuity [18]. Similarly, Roohipoor et al. reported substantial partial recoveries in parafoveal vessel density at one and three months post-removal (*p* < 0.0001 and *p* = 0.01, respectively), along with modest increases in foveal thickness. Nevertheless, these parameters remained below normative levels [30]. Nassar et al. (2024) also observed a significant increase in CFT, from 254.90 µm to 265.50 µm (*p* = 0.002), along with a notable improvement in retinal sensitivity, from 5.04 dB to 6.38 dB (*p* < 0.001), despite vascular density parameters remaining unchanged [20].

Conversely, some studies indicate limited recovery or persistent deficits. Lee et al. reported significant structural thinning in CFT (243.55 µm vs. 265.06 µm in unaffected eyes, *p* = 0.015) and a reduction in macular ganglion cell-inner plexiform layer thickness (66.13 µm vs. 78.13 µm, *p* < 0.001) after SO tamponade. Additionally, prolonged SO tamponade was correlated with increased DCP-FAZ (r = 0.3225; *p* = 0.0483) and decreased VD in the deep capillary plexus (r = −0.3588; *p* = 0.0269) [27]. Similarly, Salehi et al. found no significant changes in macular thickness, superficial and deep capillary plexus densities, or FAZ parameters over three months, indicating persistent structural and vascular stability without clear recovery [26].

### 3.6. Quality Assessment of Included Studies

Considering the observational nature of all included studies, we performed a structured methodological appraisal using the Newcastle–Ottawa Scale (NOS). The NOS scores among the 23 studies ranged from 5 to 8, with a median score of 7. Overall, 13 studies (57%) were classified as having a low risk of bias (7–8 stars), whereas 10 studies (43%) were deemed to have a moderate risk (5–6 stars). No studies were assessed at high risk (≤4 stars). The primary limitations identified pertained to the retrospective single-center design and the absence of multivariable adjustments for RRD-related confounders, including macular status at presentation, duration of detachment, PVR grade, or SO duration and viscosity. Additionally, several studies reported relatively short follow-up periods, which restricts the ability to draw definitive conclusions regarding long-term perfusion and functional outcomes. Detailed study-level NOS scores are provided in Supplementary Table S2.

Furthermore, we have extracted methodological parameters related to study design, patient populations, SO characteristics, imaging protocols, and follow-up intervals. This information is summarized in Supplementary Table S3 to aid in the interpretation of inconsistent findings across various studies.

The reporting of OCTA scan quality demonstrated inconsistencies across the included studies. Out of 23 studies, 22 specified a scan-quality metric, with 20 prespecifying a threshold, most commonly SSI > 6/10, while only five reported mean scan-quality values. All studies indicated that images of poor quality were excluded from analysis. Detailed information regarding the device, scan parameters, and quality assessment for each study is provided in Supplementary Table S4.

**Table 1:** Overview of studies assessing FAZ, Vessel Density, and Retinal Thickness after RRD surgery.

Study (Ref)	Design	N (Eyes)	Macular Status	Device and Scan Size	FAZ	SCP VD	DCP VD	Retinal Thickness	Control vs	Follow-Up	Correlation with Recovery
Jiang et al. [22]	Retrospective	19	Mac-off	Optovue 6 × 6 mm	↔	↓ at 2 w, ↔ at 12 w, ↓ at 16 w	↓ at 2–8 w, ↔ at 12 w, ↓ at 16 w	FMT ↓ from 4 to 16 w	Fellow eye	16 wks	No significant recovery
Lee & Park [28]	Retrospective	48	Mac-on/off	Topcon SS-OCTA 4.5 × 4.5 mm	↑	SCP ↔ (nasal ↓)	DCP ↓ (average, nasal, temporal)	Not evaluated	Fellow eye	6 mo	SO duration correlated with BCVA
Lee et al. [27]	Retrospective	38	Mac-on/off	Zeiss PLEX Elite SS-OCTA 3 × 3 mm	↑ DCP/SCP	SCP ↔	DCP ↓	IR thickness ↓	Fellow eye	3 mo	DCP FAZ correlated (longer SO worse)
Liu et al. [24]	Retrospective	17 (SO)	Mac-on	Optovue 3 × 3 + 6 × 6 mm	↔	SCP ↓	DCP ↔	IR thickness ↓	Fellow eye and gas	≥30 mo	—
Nassar et al. [20]	Prospective	30	Mac-off	Optovue 6 × 6 mm	↔	SCP ↔	DCP ↔	CFT ↑ post-SOR	Baseline (pre vs. post-SOR)	1 mo	Positive functional recovery
Roohipoor et al. [30]	Prospective	45	Mac-off	Optovue 3 × 3 mm	↔	SCP ↓ early, partial ↑ later (still < fellow)	DCP ↓ foveal 1–3 m	CFT ↓, partial ↑ later	Fellow eye	3 mo	Partial (foveal DCP mild recovery)
Salehi et al. [26]	Prospective	43	Mac-off	Optovue 6 × 6 mm	↔	SCP ↔	DCP ↔ (temporal deficit)	Stable	Fellow eye	3 mo post-SOR	No significant recovery
Fang et al. [25]	Prospective	29 (20 SO, 9 air)	Mac-off	Optovue 3 × 3 mm	↔	SCP ↓ SO vs. air (1 m, 3 m); ↔ within SO	DCP ↔	PFRT ↓ in SO vs. air	Gas (external control) + within-SO	3 mo	Limited (SCP only)
Ya et al. [29]	Retrospective	7	Mac-off	Optovue 6 × 6 mm	↔	SCP ↓	DCP ↔	Not evaluated	Fellow eye	~3 mo	—
Hou et al. [18]	Prospective	50	Mac-off	Zeiss AngioPlex 6 × 6 mm	—	SCP ↓ during SO, ↑ after SOR	DCP ↔	CRT ↑ post-SOR	Gas controls + fellow + baseline	3 mo post-SOR	Improvement (macular only)
Gironi et al. [23]	Retrospective	82	Mac-on/off	Canon OCT-HS100 3 × 3 mm	—	SCP ↓ & VLD ↓ (esp. Mac-off)	DCP ↔	CMT ↑, CME ↑	Fellow eye + gas	12 mo post-SOR	SO duration inversely correlated with SCP VD

Columns summarize study design, sample size, macular status (on/off), SO characteristics (viscosity and duration), surgical adjuncts, imaging device and scan size, functional assessments, and primary outcomes. The “Control vs” column specifies the comparator used in each study (fellow eye, external control such as gas/air, or within-eye baseline pre- vs. post-SOR). Arrows indicate the direction of change compared with the specified control at the reported time points (↓ = decrease, ↑ = increase, ↔ = no significant change). A dash (—) indicates not reported or not applicable.

**Table 2:** Evaluation of Subfoveal Choroidal Thickness and Choriocapillaris Changes after RRD Surgery.

Study (Ref)	Design	N (Eyes)	Tamponade	Macular Status	OCT/OCTA Characteristics	Follow-Up	Main Changes Observed	Control vs	Correlation with SO Duration/Recovery
Chen et al. [32]	Retrospective	36	SO 5000–5400 cSt	Mac-on/off	Heidelberg Spectralis EDI-OCT, CVI (luminal, stromal, total area), CT	Pre-SOR vs. ≥2 mo post-SOR	↓ CVI (*p* < 0.001), ↓ luminal area; ↔ stromal/total area, ↔ CT; partial CVI recovery post-SOR	Fellow eye + baseline (pre vs. post-SOR)	Longer SO → greater CVI reduction
Karasu et al. [33]	Prospective	70	SO 5000 cSt	Mac-off	Heidelberg Spectralis EDI-OCT, SFCT	3 mo post-SOR	↓ SFCT (*p* = 0.004), proportional to SO duration; partial ↑ post-SOR	Baseline (pre vs. post-SOR)	SO > 9 mo → significant SFCT thinning
Karimi et al. [34]	Retrospective	60 (all pseudophakic)	SO 5700 cSt	Mac-off	Heidelberg Spectralis EDI-OCT, SFCT	3 mo post-SOR	↓ SFCT (*p* < 0.001), more pronounced in prolonged SO (>6 mo); minimal recovery (~2 µm)	Fellow eye + baseline (pre vs. post-SOR)	Longer SO (>6 mo) → less SFCT recovery
Mirza et al. [35]	Prospective	24 (19 analyzed)	SO 1000 cSt	Mac-off	Heidelberg Spectralis EDI-OCT, SFCT	2 wks, 3 mo (SO in situ) + 1 mo post-SOR	SFCT: ↔ at 2 w and 3 m during SO (*p* = 0.96); ↓ at 1 m post-SOR (*p* = 0.03 vs. baseline)	Fellow eye + baseline (pre vs. post-SOR)	ΔSFCT% correlated with SO duration (rho = 0.537, *p* = 0.032)
Prasuhn et al. [36]	Retrospective before–after	19 (all pseudophakic)	SO 5000 cSt	Mac-off	Zeiss Cirrus AngioPlex 6 × 6 mm OCTA + EDI-OCT	4 wks post-SOR	↑ CCP perfusion (*p* = 0.0013); ↓ Sattler’s (*p* = 0.034) & Haller’s (*p* = 0.0402); ↔ SFCT	Baseline (pre vs. post-SOR)	Redistribution of choroidal perfusion after SOR

The table summarizes study design, sample size, macular status (on/off), SO characteristics (viscosity and duration), imaging modality (OCT/OCTA), follow-up intervals, and main structural/vascular outcomes. The column “Control vs” specifies the comparator used in each study (fellow eye, external control such as gas/air, or within-eye baseline pre- vs. post-SOR). Arrows indicate the direction of change compared with the specified control (↓ = decrease, ↑ = increase, ↔ = no significant change).

**Table 3:** Functional outcomes (microperimetry and BCVA) after RRD surgery with silicone oil tamponade.

Study (Ref)	Study Design	Phase	N (Eyes)	Macular Status	Functional Modality (Device/Parameters)	Follow-Up	Main Functional Changes	Control vs	Quality/Reliability Reporting	Correlation with Recovery Potential
Delolme et al. [37]	Observational case series	Post-op (≥6 mo after RRD repair; PPV/SB; small SO subgroup)	30	Mac-off	Microperimetry (Spectral OCT/SLO, ~28 points, ~11–12°); BCVA	Mean 23.1 ± 10.3 mo (7–37)	RS ↓ in eyes with IS/OS disruption; foveal RS ↓ with PROS thinning; BCVA not strongly associated with lesion type	Fellow eye	Clear media; OCT reviewed by two graders; fixation tracked	RS associated with IS/OS integrity and PROS thickness
Dou et al. [38]	Retrospective cohort	Post-SOR	48	Mac-off	Microperimetry (Nidek MP-3, 45 points, 12°); BCVA logMAR	Pre-SOR vs. 3 mo post-SOR	RS ↑ (2° and 6°, both *p* < 0.05); fixation rate ↑; BCVA ↔	Baseline (pre vs. post-SOR)	OCTA SSI >6; poor images excluded; central fixation required	RS/FR improvement despite DCP VD ↔; SCP VD ↑ and RNFLT ↑ post-SOR
Nagpal et al. [39]	Prospective interventional	Post-SOR	20	Mac-off	Microperimetry (Nidek MP-3, 37 stimuli, 12°); BCVA logMAR	Pre-SOR vs. 1 mo post-SOR	RS ↑ in 100% of eyes (*p* < 0.0001); BCVA ↔	Baseline (pre vs. post-SOR)	MP reliability criteria (<15% false answers); dark room; eye-tracking	Functional gain without BCVA change; early recovery signal
Nassar et al., 2024 [20]	Prospective case series	Post-SOR	30	Mac-off	Microperimetry (Optos SLO, 28 points, 11°); BCVA logMAR	Pre-SOR vs. 1 mo post-SOR	RS ↑ (*p* < 0.001); BCVA ↑ (*p* < 0.001)	Baseline (pre vs. post-SOR)	OCTA SSI > 6; poor images excluded; standardized SLO grid	ONH/RPC VD ↑ post-SOR while macular VD ↔ → function may recover independently of macular perfusion
Nassar et al., 2019 [21]	Prospective comparative cohort	Post-SOR	22	Mac-off	Microperimetry (Optos SLO, 28 points, 11°); BCVA logMAR	1 day pre-SOR vs. 1 mo post-SOR (<3 mo SO vs. 3–6 mo SO)	RS ↑ in both groups (*p* ≤ 0.008); BCVA ↑ (*p* ≤ 0.007)	Baseline (pre vs. post-SOR; two SO-duration subgroups)	MP reliability criteria; dark room; fellow eye occluded	RS gain independent of SO duration (≤6 mo)
Scheerlinck et al., 2016 [40]	Retrospective cohort	During SO and post-SOR (2 mo)	193 (SO subset 37; MP subset ~10)	Mac-on	Microperimetry (Optos OCT/SLO, 21 points, 11°); BCVA	~2 mo post-SOR; extended follow-up in subset	Distinct central 2° scotoma in eyes with unexplained VL; RS severely ↓; BCVA ↓ in VL eyes	Gas controls + baseline (pre vs. post-SOR)	OCT SSI ≥7; poor scans excluded	SO duration = only significant risk factor for unexplained VL
Scheerlinck et al., 2018 [19]	Prospective observational cohort	Post-SOR (SO) vs. post-op (gas)	40 (10/group)	Mixed (Mac-on/off)	Microperimetry (Optos OCT/SLO, 25 stimuli, 11°); BCVA	2 mo post-op	RS ↓ in SO vs. gas (Mac-on: 11.8 vs. 15.6 dB, *p* = 0.003; Mac-off: 11.6 vs. 15.0 dB, *p* = 0.037); small central scotomas only in SO groups	Gas controls	Reliability checked; poor MP scans excluded	Functional sensitivity disadvantage with SO vs. gas at 2 mo

The table presents study design, phase (during SO or post-SOR), sample size, macular status, functional assessment modality (microperimetry/BCVA), follow-up intervals, and main functional outcomes. The column “Control vs” specifies the comparator used in each study (fellow eye, external control such as gas, or within-eye baseline pre- vs. post-SOR). Arrows indicate the direction of change compared with the specified control (↓ = decrease, ↑ = increase, ↔ = no significant change).

## 4. Discussion

This literature review examined studies investigating the impact of SO tamponade on retinal and choroidal structures, vascular alterations, and related functional outcomes. Key findings revealed substantial variability in macular and peripapillary vessel densities, structural modifications within retinal and choroidal layers, and changes in visual function following SO tamponade and removal.

Retinal vascular assessments indicated a generally reduced SCP-VD following SO tamponade, though post-removal recovery was inconsistent [22,23,24]. Results regarding the DCP-VD varied notably, with studies reporting either significant reductions or minimal changes [22,24,27,28]. Analysis of the FAZ yielded mixed outcomes; significant enlargement was predominantly observed in the DCP, particularly in cases involving prolonged SO tamponade [27,28]. In contrast, other studies have noted minimal or no significant alterations in the FAZ [20,24,25,26,29,30].

Peripapillary vascular changes consistently show significant reductions in VD, closely associated with decreased RNFLT, indicating ongoing vulnerability in both retinal structure and vascular integrity [22,26,31].

Analysis of choroidal parameters indicated significant reductions in the CVI and SFCT, which demonstrated a strong correlation with prolonged durations of SO tamponade. Partial structural and perfusion recovery was observed following the removal of SO [32,33,34,35]. Notably, redistribution of perfusion among the choroidal layers after SO extraction was documented, emphasizing intricate vascular modifications [36].

Functional retinal assessments through microperimetry consistently reveal improvements in retinal sensitivity and stabilization of fixation following SO removal, notwithstanding ongoing or partial structural and vascular recoveries [20,21,37,38,39,40]. Conversely, some studies have identified significant central scotomas and persistent deficits in central retinal sensitivity, particularly after SO compared to gas tamponade [19,40].

### 4.1. Interpretation of Key Results

The substantial reductions in retinal VD related to SO tamponade indicate potential vascular impairment that may result from mechanical compression or biochemical alterations induced by SO [22,23,24,25,26]. Frequent reports of decreased VD in the SCP underscore the notable susceptibility of superficial retinal vessels to the stress caused by SO [22,23,24]. Conversely, the inconsistent findings within the DCP may be attributable either to methodological variations across studies or to intrinsic differences in ischemic tolerance between retinal layers [22,24,27,28]. Another possible explanation could reside in the closer proximity of the SO itself to the SCP than to the DCP.

However, the previous explanation is not in agreement with the choroidal vascular alterations which show a decrease in the CVI and SFCT. These observations likely reflect prolonged mechanical stress and impaired choroidal perfusion attributable to SO tamponade [32,33,34,35,36]. These structural modifications suggest vascular compromise which may lead to ischemia and subsequent remodeling or atrophy of choroidal vessels. The partial recovery observed following SO removal indicates transient and potentially reversible choroidal impairment; however, incomplete restoration underscores the risk of irreversible choroidal damage with extended periods of tamponade [32,33,34,35].

Reductions in peripapillary VD, along with associated thinning of the RNFLT, are likely attributable to ongoing mechanical compression or modifications in fluid dynamics instigated by SO tamponade [22,26,31]. The persistent correlation between diminished peripapillary VD and RNFLT thinning highlights a direct relationship, suggesting that impaired perfusion influences the integrity of the optic nerve head and visual function.

The functional improvements observed through microperimetry following the removal of SO underscore the retina’s capacity for partial functional recovery, despite the persistence of incomplete structural or vascular normalization [20,21,37,38,39,40]. These outcomes of functional recovery emphasize the clinical significance of timely SO removal in reducing persistent visual deficits and enhancing visual rehabilitation results.

Taken together, these findings indicate that prolonged SO tamponade substantially increases the risk of persistent retinal and choroidal damage. Therefore, it is crucial to tailor clinical management carefully, particularly regarding the duration of SO tamponade, to improve visual rehabilitation outcomes.

### 4.2. Integration with Existing Literature

Recent literature broadly supports our findings regarding the impact of SO tamponade on retinal and choroidal microvasculature and associated functional outcomes. Christou et al. reported consistent reductions in retinal vessel density in SO-filled eyes, predominantly within the SCP, corroborating our observations of persistent SCP vessel density reductions post-SO tamponade [3,22,23,24,25,26]. They emphasized a partial, though incomplete, recovery of these metrics post-removal, aligning closely with the patterns observed in our review [22,24,26].

Ferrara et al. emphasized considerable variability in the vessel density outcomes of the DCP, noting a pronounced loss of DCP, especially in cases involving prolonged SO tamponade, which aligns with our findings [22,27,28,41]. They also observed enlarged FAZs, primarily within the DCP, thereby supporting the conclusions of our review that associate extended SO duration with FAZ enlargement in the DCP [27,28]. Nonetheless, Ferrara et al. additionally acknowledged that some studies reported stable FAZ parameters, a finding consistent with certain studies included in our review that demonstrated no significant changes in FAZ [24,25,26,29,30].

Regarding choroidal alterations, Chen et al. documented notable decreases in the CVI during SO tamponade, correlating them with an extended duration of tamponade, which is in agreement with the observations of our review [32,33,34,35,42]. Chen’s findings notably support our conclusion that reductions in choroidal thickness and CVI may partially recover following SO removal, but they frequently remain affected [35,36].

Januschowski et al. emphasized notable decreases in peripapillary vessel density and RNFLT during and after SO tamponade, which corroborates our findings of enduring peripapillary vascular and structural impairments [22,26,31,43]. These researchers established robust correlations between RNFLT thinning and reductions in vessel density, underscoring the structural–functional interdependence identified in our review.

Furthermore, Chen et al.’s systematic review corroborated the existence of persistent impairments in retinal sensitivity, along with subsequent improvements following SO removal, which is consistent with our microperimetry findings [37,38,39,40,42]. They also emphasized variability in recovery rates, thereby supporting our review’s conclusions regarding partial and inconsistent functional recovery, which are contingent upon the duration of SO and the initial retinal condition.

Overall, the recent literature predominantly concurs with the findings of our review, consistently recognizing substantial vascular and structural changes associated with SO tamponade, and highlighting variability in outcomes attributable to methodological differences and individual patient-related factors. This consensus underscores the vital importance of prompt SO removal and meticulous clinical follow-up to prevent potential adverse outcomes.

### 4.3. Structure–Function Relationships and Perfusion Correlations

Numerous investigations have examined the correlations between structural modifications in the retina and choroid and the associated functional results subsequent to SO tamponade.

Retinal sensitivity, as evaluated through microperimetry, exhibited a significant correlation with photoreceptor integrity. Delolme et al. established a direct association between diminished retinal sensitivity and photoreceptor abnormalities, notably in areas with disrupted IS/OS junctions (*p* = 0.029) [37]. Similarly, Dou et al. documented an improvement in retinal sensitivity following SO removal, which was closely associated with enhanced fixation stability and reduced tamponade durations, thereby underscoring the functional significance of these structural parameters [38].

Regarding choroidal perfusion, Mirza et al. documented a significant reduction in SFCT subsequent to SO removal, with a positive correlation to the duration of tamponade, likely indicative of persistent mechanical stress [35]. Similarly, Chen et al. observed significant decreases in the CVI during SO tamponade, primarily attributable to reductions in the luminal area, with partial recovery noted following removal, thus underscoring the potential reversibility of ischemic effects [32].

Results concerning retinal VD following SO removal exhibited considerable variability. Hou et al. reported significant enhancements in SCP-VD, which showed a positive correlation with improvements in visual acuity, thereby emphasizing functional recovery associated with vascular regeneration [18]. Conversely, Lee et al. observed enduring reductions in DCP-VD, strongly linked to prolonged SO tamponade, as evidenced by significant enlargement of the FAZ (*p* < 0.001). Such disparities highlight possible differences in methodology, duration of SO tamponade, or baseline retinal ischemia [27].

Peripapillary changes documented by Jiang et al. demonstrated a significant reduction in RPC-VD, with a strong correlation to RNFL thinning (r = 0.636, *p* = 0.003). Continued decreases in RPC VD following SO removal, as reported by Salehi et al., suggest persistent vascular impairment and directly implicate compromised perfusion as a potential causative factor in structural optic nerve damage and visual field deficits [26,31].

The FAZ metrics demonstrated heterogeneous outcomes. Lee and Park recorded significant enlargement primarily within the DCP (*p* = 0.002), particularly in cases of macula-off RRDs, which may suggest severe deep retinal ischemia [28]. Conversely, other studies reported consistent FAZ parameters, indicating possible methodological differences or variations in patient populations [20,26,29,30].

In summary, the consistent correlations between structural and perfusion impairments with functional outcomes underline the clinical relevance of timely SO removal, emphasizing the need to minimize irreversible retinal and choroidal damage. The observed variability across studies further highlights the importance of standardized assessment protocols and additional research to clarify specific factors influencing these clinical outcomes.

### 4.4. Clinical Significance

The findings of this review bear significant implications for clinical practice. The identified vascular and structural impairments associated with SO tamponade—including reduced retinal and peripapillary VD, RNFL thinning, and diminished choroidal thickness—underline the potential risks linked to prolonged SO retention [22,23,24,25,26,31,32,33,34,35,36]. These results emphasize the critical importance of timely SO removal, ideally within three to six months, in order to minimize irreversible structural damage and maximize the potential for visual recovery [18,20,21,26,30,34,35,36].

Functional assessments utilizing microperimetry further corroborate this perspective by demonstrating notable enhancements in retinal sensitivity following SO removal, notwithstanding incomplete structural recovery. These findings highlight the clinical significance of prompt intervention in safeguarding functional visual outcomes [20,21,37,38,39,40].

Furthermore, routine postoperative monitoring using OCTA and microperimetry can identify subtle yet clinically significant changes in retinal and choroidal structures and perfusion. These advanced diagnostic methods enable clinicians to promptly recognize structural–functional correlations, thereby supporting more precise management strategies and patient counseling.

In the light of these insights, healthcare professionals are advised to implement an individualized management strategy, meticulously balancing the risks linked to extended tamponade with the patient’s specific clinical requirements. The early identification of subtle retinal alterations and the timely removal of the SO can substantially improve prognosis and optimize long-term visual results.

### 4.5. Sources of Heterogeneity and Inconsistent Findings

Across the 23 included studies, the reported vascular and functional outcomes after SO tamponade in RRD were not always consistent. Some studies demonstrated significant reductions in SCP and/or DCP vessel density, others reported no change, while a few described partial recovery following SO removal. Likewise, FAZ size was found to be enlarged in certain series and stable in others, and functional outcomes such as microperimetry and BCVA showed variable improvements. These apparent contradictions are best explained by methodological heterogeneity rather than by true biological discrepancies.

The primary sources of variability encompassed study design (retrospective vs. prospective), patient populations (macula-on vs. macula-off, varying durations of detachment, presence of PVR), and SO characteristics (1000 vs. 5000 cSt, duration of tamponade ranging from 3 months to >9 months). Imaging protocols also exhibited differences, with the majority of series utilizing Optovue devices, while others employed Zeiss, Canon, or Topcon platforms, and scan sizes varied from 3 × 3 mm to 6 × 6 mm. Additionally, different segmentation strategies and artifact correction methods further complicated comparisons across studies. Moreover, follow-up intervals displayed considerable variation, extending from 1 month to over 3 years, and outcome measures lacked uniformity: some studies focused solely on BCVA, others incorporated microperimetry, and only a minority attempted to correlate functional measures with perfusion metrics.

These sources of heterogeneity elucidate much of the inconsistency observed across studies. Instead of indicating fundamentally conflicting biological effects of SO, they emphasize the methodological diversity inherent in the available literature. Nevertheless, several consistent patterns are evident: reduced perfusion, particularly in the SCP, during SO tamponade; partial but frequently incomplete recovery after SO removal; more pronounced structural and vascular changes associated with prolonged tamponade duration and higher viscosity oils; and variable functional recovery, which generally correlates with structural and perfusion integrity.

Image quality constitutes an additional source of heterogeneity in OCTA-based vessel density measurements. Factors such as reduced visual acuity, unstable fixation, and media opacities during SO tamponade can impair scan quality, resulting in motion or projection artifacts that may artificially decrease VD. Conversely, after SO removal, improvements in optical media and fixation stability may enhance image quality, potentially simulating an apparent increase in perfusion rather than indicating true biological recovery. In our analysis, although 22 out of 23 studies specified a scan-quality metric and 20 pre-established a threshold, only five reported average quality values. The limited and inconsistent documentation of scan quality (see Supplementary Table S4) impedes cross-study comparability and may partly explain the discrepancies observed within the literature.

### 4.6. Limitations of Current Evidence

The reviewed studies demonstrate significant methodological variability, especially concerning differences in OCTA protocols, inconsistent definitions, and diverse measurements of retinal and choroidal parameters. Patient demographics, clinical conditions, and baseline characteristics also vary considerably, potentially leading to substantial heterogeneity and restricting the capacity for direct comparison and generalization of the results. Follow-up durations exhibit considerable variation across studies, further complicating evaluations of long-term vascular, structural, and functional outcomes.

Most studies included in this review had a low to moderate risk of bias, mainly due to retrospective design, short follow-up periods, and limited control over RRD-related confounders. Therefore, conclusions from OCTA and microperimetry studies after SO tamponade should be viewed cautiously, although the overall trends seem consistent across studies.

### 4.7. Recommendations for Future Research

Future research should prioritize well-designed, prospective longitudinal studies incorporating standardized OCTA and microperimetry protocols. Implementing uniform definitions and consistent measurement methodologies for retinal and choroidal parameters across studies is crucial for enhancing the comparability of results. Recruiting larger, homogeneous patient cohorts would substantially reduce variability, improve the validity of conclusions, and enhance the generalizability of findings. Addressing specific gaps, such as clarifying the precise influence of SO tamponade duration on retinal, choroidal, and functional recovery outcomes, remains essential. Prospective studies with regular, standardized follow-up intervals post-SO removal would be highly beneficial for determining optimal intervention timing and guiding evidence-based clinical decision-making.

## 5. Conclusions

SO endotamponade effectively facilitates retinal reattachment; however, it is associated with significant structural and vascular alterations in the retina and choroid. Studies consistently document reduced macular and peripapillary VD, choroidal thinning, and retinal nerve fiber layer deterioration during SO tamponade, correlating with measurable losses in retinal sensitivity detected via microperimetry. Although partial recovery of vascular and functional parameters typically occurs following SO removal, many patients fail to achieve complete restoration of baseline visual function or perfusion levels. Collectively, these findings highlight the importance of employing advanced imaging techniques, such as OCTA, alongside functional evaluation tools like microperimetry, to effectively monitor and manage patients following RRD repair. Timely SO removal, combined with additional therapeutic measures aimed at supporting retinal and choroidal microvascular health, should be prioritized in clinical practice. Future prospective studies with standardized methodologies are mandatory to establish clear guidelines, improve prognostic evaluations, and optimize visual recovery for these patients.

## Supplementary Materials

The following supporting information can be downloaded at: https://www.mdpi.com/article/10.3390/diagnostics15192422/s1, Supplementary Material S1: Data sources and PubMed search strategy; Supplementary Table S2: Quality assessment of included studies (OCTA and microperimetry, NOS); Supplementary Table S3: Methodological details and sources of heterogeneity; Supplementary Table S4: Scan-quality reporting across included studies.

## Abbreviations

RRD	Rhegmatogenous retinal detachment
SO	Silicone oil
OCTA	Optical coherence tomography angiography
FAZ	Foveal avascular zone
VD	Vessel density
SCP	Superficial capillary plexus
DCP	Deep capillary plexus
CFT	Central foveal thickness
SFCT	Subfoveal choroidal thickness
CVI	Choroidal vascularity index
SVD	Superficial vessel density
SPD	Superficial perfusion density
GCL-IPL	Ganglion cell layer–inner plexiform layer
Temp/Inf	Temporal/inferior
dB	Decibel (unit for retinal sensitivity in microperimetry)
mo	Months
w	Weeks
