# Supplementary material for: The Role of OCTA and Microperimetry in Revealing Retinal and Choroidal Perfusion and Functional Changes Following Silicone Oil Tamponade in Rhegmatogenous Retinal Detachment: A Narrative Review
Source: Diagnostics (Basel). 2025 Sep 23;15(19):2422. doi: 10.3390/diagnostics15192422 (PMC12523867; doi:10.3390/diagnostics15192422)
Supplement: Supplementary file 1 [file diagnostics-15-02422-s001.zip › Supplementary Material 1.pdf]

Supplementary Material 1

Data Sources and Search Strategy

PubMed: 138 articles

- **Vascular changes – OCTA:**

(("Retinal Detachment"[Mesh]) OR ("Rhegmatogenous Retinal Detachment"[All Fields])) AND (("Silicone Oils"[Mesh]) OR ("Silicone Oil"[All Fields]) OR ("Gas Tamponade"[All Fields])) AND (("Optical Coherence Tomography"[Mesh]) OR ("Optical Coherence Tomography Angiography"[All Fields]) OR ("OCTA"[All Fields]) OR ("Vessel Density"[All Fields]) OR ("Choroidal Vascularity Index"[All Fields]) OR ("Macular Perfusion"[All Fields]))

PubMed : 18 articles

- **Functional assessment – microperimetry:**

(("Retinal Detachment"[Mesh]) OR ("Retinal Detachment"[All Fields])) AND (("Silicone Oils"[Mesh]) OR ("Silicone Oil"[All Fields])) AND (("microperimetry"[All Fields]) OR ("Retinal Sensitivity"[All Fields]) OR ("Fixation, Ocular"[Mesh]) OR ("Fixation Stability"[All Fields]))
